# Arachidonic acid supplementation does not affect *N*-methyl-*N*-nitrosourea-induced renal preneoplastic lesions in young Lewis rats

**DOI:** 10.3892/ol.2013.1162

**Published:** 2013-01-30

**Authors:** KATSUHIKO YOSHIZAWA, YUKO EMOTO, YUICHI KINOSHITA, AYAKO KIMURA, NORIHISA UEHARA, TAKASHI YURI, NOBUAKI SHIKATA, TOMOHITO HAMAZAKI, AIRO TSUBURA

**Affiliations:** 1 Department of Pathology II, Kansai Medical University, Moriguchi, Osaka 570-8506;; 2 Division of Pathology, Kansai Medical University Takii Hospital, Moriguchi, Osaka 570-8507;; 3 Institute of Natural Medicine, University of Toyama, Toyama 930-0194, Japan

**Keywords:** arachidonic acid, fatty acids, *N*-methyl-*N*-nitrosourea, kidney, mesenchymal tumor, nephroblastoma, rats

## Abstract

Arachidonic acid (AA) is naturally found in human breast milk. AA, together with docosahexaenoic acid, is commonly added as a functional food ingredient to commercial infant formula worldwide, in accordance with the international standards of Codex Alimentarius. However, few studies of the possible renal carcinogenic effects of AA supplementation during neonatal life have been performed. The effect of dietary AA supplementation in dams during gestation and lactation was investigated on *N*-methyl-*N*-nitrosourea (MNU)-induced preneoplastic lesions in the kidneys of young Lewis rats. Dams were fed a 2.0% AA diet or a basal diet (<0.01% AA). At birth (postnatal day 0), male and female pups received a single intraperitoneal injection of 35 mg/kg MNU or vehicle. Renal morphology was examined after 7, 14, 21, 28 and 60 days. Histopathologically, renal preneoplastic lesions, such as nephroblastomatosis and mesenchymal cell proliferation, were found on day 60 in both the MNU-treated groups. There was no significant difference in lesion incidence of 38% in the basal diet group and 31% in the AA diet group. In conclusion, an AA-rich diet for dams during gestation and lactation does not modify MNU-induced renal preneoplastic lesions in their offspring.

## Introduction

Renal cancer is the 15th most common type of cancer in the world and causes the deaths of more than 91,000 individuals every year (1). Wilms' tumor (nephroblastoma) accounts for almost 6% of all pediatric cancers and more than 95% of all kidney tumors in children (2). It is an embryonal malignancy that afflicts 1 in 10,000 children. The principle risk factors for renal cancer include inherited germline mutations. A number of loci involved in the development of Wilms' tumor have been characterized, and the key locus is *WT1*, a tumor suppressor gene located on chromosome 11p (1).

*N*-methyl-*N*-nitrosourea (MNU), a direct-acting alkylating agent that interacts with DNA, is toxic and carcinogenic to the breast and pancreas as well as the immune, hematopoietic, reproductive, dental, gastrointestinal, nervous and sensory systems (3–5). Nitrosourea compounds including MNU have carcinogenic potency in the kidney of rats (6–8), and MNU induces mesenchymal tumors and nephroblastomas in rats (9,10). The formation and persistence of DNA adducts such as O^6^-methylguanine in renal cortical tubular cells and mesenchymal interstitial cells are related to the tumor development induced by alkylating agents (8).

Arachidonic acid (AA; 20:4n-6) is a polyunsaturated fatty acid present in the phospholipids of cell membranes (11). AA in the human body comes from dietary sources such as egg yolk, or it is synthesized from linoleic acid (12). AA is naturally found in human breast milk. AA, together with docosahexaenoic acid, is commonly added as a functional food ingredient to commercial infant formula worldwide, in accordance with the international standards of Codex Alimentarius (13). Omega-3 fatty acids, such as docosahexaenoic acid, affect the growth of several cancers (14) and AA has been reported to affect carcinogenesis in certain organs. AA promotes the growth of tumors from an orthotopically transplanted breast cancer cell line (KPL-1) in female athymic BALB/c mice, urinary bladder tumors in a medium-term multi-organ rat carcinogenesis study (15), and preneoplastic lesions of the exocrine pancreas in an MNU-treated rat model (5). AA metabolites and enzymes are associated with renal tumors and related disease; cyclooxygennase-2 expression is associated with renal carcinoma (16,17), and prostaglandin E2 is associated with paraneoplastic hypercalcemia in nephroblastoma (18,19). The aim of the present study was to elucidate the effect of prenatal and postnatal dietary AA on MNU-induced renal carcinogenesis in young Lewis rats.

## Materials and methods

### Animal procedures

The study protocol and all animal procedures were approved by the Animal Care and Use Committee of Kansai Medical University and were in accordance with the guidelines for animal experimentation at Kansai Medical University. Sixteen female SPF/VAF rats (LEW/CrlCrlj) that were 10 weeks old and one-week pregnant were purchased from Charles River Japan (Yokohama, Japan). Rats were maintained in specific pathogen-free conditions and had free access to water and CE-2-modified diets containing different doses of AA. Animals were housed in plastic cages with paper-chip bedding (Paper Clean, SLC, Hamamatsu, Japan) in an air-conditioned room at 22±2°C and 60±10% relative humidity with a 12 h light/dark cycle. The illumination intensity in the cages was less than 60 lux. Offspring were culled to a maximum of 10 per dam, and the dams were maintained on their respective diets during the 21-day lactation period. During a post-weaning period of up to 60 days, the offspring were maintained on a CE-2 diet. A total of 115 male and female pups were used in this study. Four to ten rats were sacrificed at each time point (7, 14, 21, 28 and 60 days), and there were similar numbers of males and females in each dietary group.

### Chemical and dose formulation

**Figure 1:**
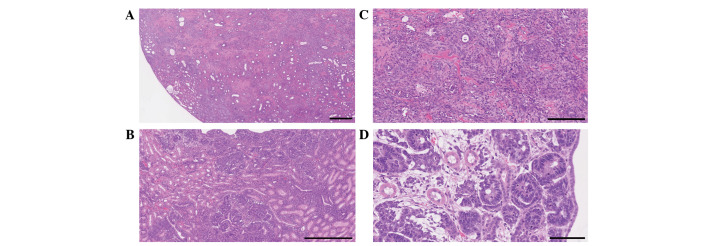
Preliminary experiment: Renal tumors in rats treated with 50 mg/kg *N*-methyl-*N*-nitrosourea (MNU) at birth (day 0). Rats were fed commercial CE-2 diet containing 0.006% arachidonic acid. (A) Sixteen weeks after MNU treatment, mesenchymal tumor exhibits non-capsulated sarcoma and entraps normal renal tubules. Hematoxylin and eosin (HE) staining, bar=500 mm. (B) Higher magnification of (A). Spindle tumor cells produced particularly mature collagen. HE staining, bar=200 mm. (C) Nephroblastoma characterized by the presence of primitive tubular and glomerular structures. HE staining, bar=500 mm. (D) Higher magnification of nephroblastoma. Note the presence of primitive proliferative glomerular structures and entrapped normal renal tubules. HE staining, bar=100 mm.

MNU was obtained from Sigma-Aldrich (St. Louis, MO, USA) and was kept at −80°C in the dark. The MNU solution was dissolved in physiologic saline containing 0.05% acetic acid immediately prior to use. MNU (35 mg/kg) or vehicle (physiological saline containing 0.05% acetic acid) was administered by intraperitoneal (i.p.) injection. In our preliminary experiment, mesenchymal tumors and nephroblastoma developed in 10 and 3 rats, respectively, of the 14 surviving rats that were treated with 50 mg/kg MNU at birth, respectively (Fig. 1). However, almost 50% of the rats died, and all surviving female rats developed mammary cancers with severe hematotoxicity. Therefore, 35 mg/kg MNU was selected as a non-lethal lower dose without the incidence of mammary cancers in the present short-term study.

### Arachidonic acid-supplemented diet

As in the previous study, the AA-supplemented diet was formulated by CLEA Japan (5,15). AA was purchased from Cargill Alking Bioengineering (Wuhan and Hubei, China). The diet with 2.0 w/w% AA was semi-purified based on the modified CE-2 formulation (CLEA Japan, Tokyo, Japan). The basal diet consisted of modified CE-2. Gas chromatographic analyses of the fatty acid composition of the diets are described in a previous study (5). The total fatty acid volumes were 47.20, 86.75 and 126.63 mg/mg of diet for the CE-2 diet (0.006 w/w% AA), basal diet (0.008 w/w% AA), and 2.0% AA diet, respectively. The diets were stored at 4°C to prevent lipid oxidation before use.

### Experimental procedures

**Figure 2:**
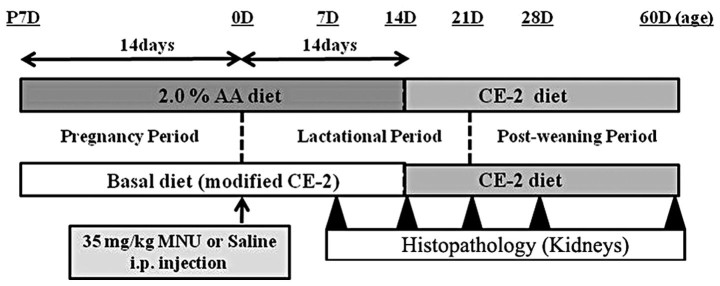
Experimental protocol. AA, arachidonic acid; HE, hematoxylin and eosin; i.p., intraperitoneal; MNU, *N*-methyl-*N*-nitrosourea.

Male and female Lewis rats were exposed to the basal or experimental diet (2.0% AA) from fertilization to sacrifice. At birth (0 days of age), the rats received an i.p. injection of vehicle (physiological saline) or 35 mg/kg MNU (Fig. 2). At 7, 14, 21, 28 and 60 days after MNU or vehicle treatment, rats were anesthetized with isoflurane (Forane^®^; Abbot Japan, Tokyo, Japan) and sacrificed by exsanguination via aortic transection. During the experiment, all pups were observed daily for clinical signs of toxicity and were weighed at the time of MNU treatment and on the day of sacrifice. Both kidneys were removed at the time of sacrifice and complete necropsies were conducted on all animals to check for systemic toxicities induced by AA supplementation. Food consumption and body weight of the dams were measured once per week to estimate the actual dosage of AA during the pregnancy and lactation periods.

### Histopathological examination

Renal tissues were fixed overnight in 10% neutral buffered formalin, embedded in paraffin, sectioned at a thickness of 4 mm and stained with hematoxylin and eosin (HE). Histopathological evaluation was performed by a toxicologic pathologist certified by the Japanese Society of Toxicologic Pathology and/or the International Academy of Toxicologic Pathology (K.Y. and A.T.). Histopathological terminology and diagnostic criteria of rodent renal neoplastic lesions were in accordance with the guidelines of the International Harmonization Nomenclature and Diagnostic Criteria for Lesions in Rats and Mice Project (20). Nephroblastoma, known as Wilms' tumor in humans, originates from the metanephric blastema. It is characterized by discrete clusters of highly basophilic blast cells surrounding mature ducts and organoid differentiation as epithelial rosettes, primitive basophilic tubules, attempted glomerulus formation or mature epithelial ducts (20,21). Nephroblastomatosis is a small, solitary, basophilic cell mass consisting of densely crowded blast cells with ill-defined cytoplasm and basophilic nuclei and a few signs of early organoid differentiation into epithelial rosettes (20,22,23). Nephroblastomatosis has the potential to develop into a nephroblastoma as it grows, and is regarded as a preneoplastic lesion of nephroblastoma (24). Mesenchymal tumors originate from foci of atypical fibroblast-like cells in the interstitium of the outer stripe of outer medulla, similar to a renal tumor of infancy described as congenital mesoblastic nephroma (22,25,26). Mesenchymal tumors are characterized by heterogeneous connective tissue cell composition with a predominance of spindle cells, primitive mesenchyme, and smooth muscle fibers and occasional rhabdomyoblasts, striated muscle, cartilage, osteoid or hemangiosarcoma-like areas (20,21). Renal mesenchymal tumors are frequently misdiagnosed as nephroblastomas due to the pre-existing tubules that survive within the tumor tissue and which can become hyperplastic and/or metaplastic (9,24). Mesenchymal cell proliferation is defined as small and solitary foci of atypical fibroblast-like cells, regarded as preneoplastic lesions and early stage mesenchymal tumors (6).

### Statistical analysis

The incidence of renal preneoplastic lesions was analyzed using the χ^2^ test. The results presented include comparisons between rats fed a basal diet and rats fed an AA-supplemented diet in the MNU-treated groups. P<0.05 was considered to indicate a statistically significant result.

## Results and Discussion

No mortalities occurred and no clinical signs or symptoms related to any treatment were evident in any of the pups or dams during the experimental period. None of the pups developed mammary tumors. The 2.0% AA diet did not influence food consumption in dams during the experimental period. During the pregnancy and lactation periods, the AA intake of dams was 6.3 and 8.5 mg/kg/day in the basal diet group and 1,477 and 1,876 mg/kg/day in the 2.0% AA group, respectively. The 2.0% AA diet did not influence body weight gain (the growth rate) in pups or cause weight changes in dams with or without MNU treatment. The growth rate in MNU-treated pups tended to be lower than that in vehicle-treated pups, however (data not shown).

**Figure 3:**
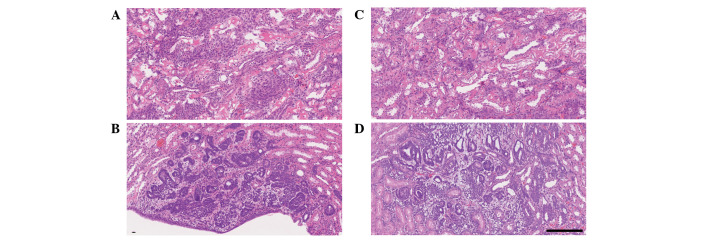
Renal morphology in rats treated with 35 mg/kg *N*-methyl-*N*-nitrosourea (MNU) at birth (day 0). Small area of mesenchymal cell proliferation in a 60-day-old rat that was fed the basal diet containing (A) 0.008% and (B) 2.0% arachidonic acid; the production of mature collagen is observed (A,B). Small area of nephroblastomatosis in a 60-day-old rat that was fed the basal diet containing (C) 0.008% and (D) 2.0% arachidonic acid; islands of blastemal cells and associated primitive tubule formation are observed (C,D). Hematoxylin and eosin (HE) staining, bar=200 mm.

The incidence of preneoplastic renal lesions is shown in Table I. Macroscopic nodular lesions were not detected in any group. In the vehicle-treated rats with or without the AA-rich diet, no proliferative lesions were observed at any time point. In contrast, single or multifocal preneoplastic lesions occurred in the basal diet-fed rats 60 days after MNU treatment (38% incidence, 3 lesions in 8 kidneys); mesenchymal cell proliferation was detected histopathologically in 2 kidneys (Fig. 3A) and nephroblastomatosis in 1 kidney (Fig. 3C). The AA-rich diet-fed rats also developed preneoplastic lesions 60 days after MNU treatment (31% incidence, 5 lesions in 16 kidneys); mesenchymal cell proliferation was detected in 2 kidneys (Fig. 3B) and nephroblastomatosis in 3 kidneys (Fig. 3D). There was no significant difference in lesion incidence between the MNU-treated rats fed a basal diet and the MNU-treated rats fed an AA-rich diet. The multiplicity of these lesions was not different between MNU-treated rats with or without AA supplementation. In these lesions, the normal tubules were entrapped and there was no evidence of capsulation. Tubular tumors were not observed in any group at any time point. Pelvic dilation was detected in some rats of each group at any time point, which is characteristic of spontaneously occurring lesions in this strain (data not shown).

The known risk factors include therapeutic doses of ionizing radiation and inherited and genetic alterations. Together they are estimated to account for 5–10% of childhood cancers (27). Nephroblastoma accounts for 95% of kidney malignancies during childhood (27). Children are exposed to potentially carcinogenic chemicals, such as pesticides, from use in homes, schools and gardens and through contaminated food and drinking water. Parent exposure during the child's gestation or even preconception may also be important. The household or occupational use of pesticides increases the risk of renal tumors in children (28). Tea or coffee consumption and certain parental occupations have been consistently associated with this type of tumor (27). The main purpose of the present study was to determine whether increased levels of AA during gestation and lactation proportionally enhance the development of renal preneoplastic lesions in MNU-treated rat pups. Renal morphology in rats treated with 35 mg/kg MNU showed nephroblastomatosis and mesenchymal cell proliferation in those fed a basal or AA-rich diet (2.0% AA). These results suggest that the increased incidence of these lesions is an early indicator of renal carcinogenesis induced by chemicals (6,24). The results demonstrate that 2.0% AA did not have any morphological effect on renal preneoplastic lesions. Recently, AA showed no promoting effects on kidneys in a rat medium-term multi-organ carcinogenesis model with 5 carcinogens including MNU (29).

In a study by Sharma *et al*, 50 mg/kg MNU i.p. injections 2 and 4 days after birth induced renal tumors at between 4 and 8 months of age. Renal tumors occurred in 63 of 140 kidneys (45%); there were 29 mesenchymal tumors, 18 nephroblastomas and 16 other tumors. The incidence of mortality, mammary tumors and hematotoxicity in this model was not reported, however (10). In this preliminary study, a single injection of 50 mg/kg MNU given to Lewis rats at birth induced a high incidence of mortality and mammary cancers with severe hematotoxicity. Rats are most sensitive to the induction of renal tumors when alkylating agents are administered at the early weeks after birth, when the rate of cell division in the immature kidney is the highest (10,22). Like this experimental protocol, a short-term study (60 days) with 35 mg/kg MNU as a non-lethal lower dose without the incidence of mammary cancers, may therefore be extremely useful for testing the promotion, progression or inhibitory effects of chemical and physical agents on cell proliferation and transformation in rat kidneys.

The AA intake by Japanese infants via breast milk is ∼14.3 mg/kg/day (29). The 2.0% AA diets in this present study provide an AA dose of 1,477 mg/kg/day during pregnancy and 1,876 mg/kg/day during lactation, representing ∼103- and 131-fold, respectively, the amount consumed by human infants. Taken together, the results indicate that an AA-enriched diet in the prenatal and postnatal periods is unlikely to cause renal carcinogenesis in human infants. In conclusion, an AA-rich diet in dams during gestation and lactation does not modify MNU-induced renal preneoplastic lesions in young rats. Further studies with other animal models are necessary to fully elucidate the effects of AA on renal carcinogenesis.

## Figures and Tables

**Table I:** Incidence of renal preneoplastic lesions induced by MNU.

Treatment	Food	Days after MNU treatment				
		7	14	21	28	60
Vehicle	Basal^b^	0 (0/12)^d^	0 (0/12)	0 (0/10)	0 (0/10)	0 (0/20)
	AA^c^	0 (0/12)	0 (0/12)	0 (0/10)	0 (0/10)	0 (0/10)
MNU^a^	Basal	0 (0/12)	0 (0/12)	0 (0/10)	0 (0/10)	38 (3/8^e^)
	AA	0 (0/12)	0 (0/12)	0 (0/10)	0 (0/10)	31 (5/16^f^)^g^

a 35 mg/kg *N*-methyl-*N*-nitrosourea (MNU) was injected intraperitoneally at birth (day 0);

b The basal diet contained 0.008% arachidonic acid (AA);

c The AA diet contained 2.0% AA;

d The percentage of incidence (number of kidneys with neoplastic lesions/total number of kidneys examined) is indicated;

e Nephroblastomatosis was detected in 1 kidney and mesenchymal cell proliferation was detected in 2 kidneys;

f Nephroblastomatosis was detected in 3 kidneys and mesenchymal cell proliferation was detected in 2 kidneys;

g no significant difference between basal diet-fed rats in MNU-treated groups.
